# Editorial: Emerging talents in reproduction: 2022

**DOI:** 10.3389/fendo.2024.1413251

**Published:** 2024-06-07

**Authors:** Ali Abbara, Alexander N. Comninos

**Affiliations:** ^1^ Section of Endocrinology & Investigative Medicine, Imperial College London, London, United Kingdom; ^2^ Department of Endocrinology, Imperial College Healthcare National Health Service (NHS) Trust, London, United Kingdom

**Keywords:** reproduction, polycystic ovary syndrome, emerging leaders, pregnancy, bone health, heat stress, assisted conception

For many years, research in reproductive health, and women’s health in particular, has been under-served. This special issue of *Frontiers in Endocrinology* encouraged *Emerging Talents* working in the field of *Reproduction* to submit articles of importance to reproductive health to support their journey in becoming future research leaders in reproduction. This endeavour is fundamental to redressing the neglect in research in reproductive health but also for driving better care for patients suffering from reproductive disorders.

The commonest endocrine disorder affecting premenopausal women is polycystic ovary syndrome (PCOS) affecting 8-13% of women. The experiences of women with PCOS were explored by Mun Lau et al. who conducted a systematic review examining lived-experiences of women living with PCOS and highlighted the need for a holistic approach. Melson et al. conducted a systematic review examining different models of care for women living with PCOS across the globe. The global digital impact of ‘PCOS awareness month’ was evaluated by Malhotra et al., who provided evidence for the positive effect of such events. Indeed, social media is increasingly recognised to have a major impact on the public’s awareness and knowledge of health issues, which can be associated with both risks and benefits depending on the source of information. Accordingly, Elhariry et al. evaluated the Top 100 influencers providing content on social media. Not all women with PCOS have all features of PCOS, leading to different subtypes. Indeed, Mills et al. evaluated the impact of individual components forming PCOS and different subtypes on bone health and the risk of osteoporosis.

With respect to obstetric health, Yüzen et al. reviewed the effect of the environment on pregnant women highlighting the potential impact of heat stress on maternal and foetal health. The use of progestins to prevent a premature rise in luteinising hormone (LH) during assisted conception cycles has been increasingly studied. Lin et al. conducted a systematic review comparing this approach to standard protocols.

Despite very recent publication dates, these seven articles have already amassed 46 citations speaking to the impact of this special issue. Therefore, as editors, we hope that this special issue provides a platform to promote emerging leaders in the field of reproduction endorsing the quality of their work, and we look forward to viewing the impact of their future work in the field.

## Author contributions

AA: Writing – original draft, Writing – review & editing. AC: Writing – original draft, Writing – review & editing.

